# Water and elevation are more important than burn severity in predicting bat activity at multiple scales in a post-wildfire landscape

**DOI:** 10.1371/journal.pone.0231170

**Published:** 2020-04-08

**Authors:** Clarissa A. Starbuck, Erin S. Considine, Carol L. Chambers

**Affiliations:** School of Forestry, Northern Arizona University, Flagstaff, Arizona, United States of America; Museu de Ciències Naturals de Granollers, SPAIN

## Abstract

Bats are among the most widespread mammals on Earth, and are subject to habitat change, loss, and other disturbances such as fire. Wildfire causes rapid changes in vegetation that affect habitat use. However, the spatial scale at which these changes affect bats depends on their use of habitat elements. Three years post wildfire, we assessed how burn severity, water, landform type, elevation, vegetation type, and roads affected use by bats of a forest landscape at multiple spatial scales. We deployed acoustic detectors at randomly selected locations within a 217,712 ha wildfire boundary in Arizona. We classified echolocation calls to species or group and calculated an activity index by adjusting the calls per hour. We conducted a multi-scale analysis of landscape structure and composition around each location from a 90 to 5760 m radius. No scale was selected preferentially by any species or group. Stream density and elevation range were more important predictors for species groups than burn severity. When burn severity was a predictor, agile species had higher activity in areas that were unburned or had low severity burn. A heterogeneous landscape composed of high, medium, and low burn severity patches within a forest altered by large wildfires provided habitat for different bat species, but water density and range in elevation were more important for predicting bat habitat use than fire severity in this arid landscape. More than one spatial scale, representing local to landscape levels, should be considered in managing habitat for bats. In arid areas, such as the western United States, maintaining reliable water sources is important for bats. Managing these factors at multiple spatial scales will benefit bat species with different wing morphologies, echolocation call types, and habitat selections.

## Introduction

The diversity of bats, with their nocturnal, volant, and cryptic behaviors, make this taxon challenging to study. Yet bats are among the most widespread mammals on Earth [1], and are subject to habitat change, loss, and other disturbances. A disturbance, such as fire, causes rapid changes in vegetation that affect habitat use by bats.

A warming climate with snowmelts occurring earlier in the year compared to historic snowmelts increases fire activity in forests in the western United States [2], and the topography of the southwest increases the probability of high severity fire in this region [3]. An increase in tree density in southwestern ponderosa pine (*Pinus ponderosa*) forests caused by less frequent low-severity fires has led to wildfires of increased severity and size [4–6].

An increase in large, severe wildfires can have differing effects on wildlife. In western meadows and forests, Horncastle et al. [7] found that small mammal occupancy was not affected by fire in Arizona, but Borchert et al. [8] found different responses from small mammal species in southern California. Low to moderate severity fire may help maintain habitat for California spotted owl (*Strix occidentalis occidentalis*; [9]), but the predicted increase of high severity fire may also be detrimental to their nesting habitat [10], so the effects of wildfire on spotted owls is still uncertain [11].

Fire can have positive or negative effects on bats that use forests for roosting or foraging. Fire can provide roosting habitat for bat species either by creating dead trees with cavities or forest gaps that increase solar radiation to roost trees [12]. Increased solar radiation improves conditions inside the roost for juvenile bats by increasing the temperature during summer [13–15]. However, large stand-replacing fires can also remove roosts from a landscape [16].

Low intensity, low severity fire, such as prescribed fire, had a positive or no effect on bat activity [12, 17, 18], but this depended on the bat species [19–21]. Armitage and Ober [21] and Inkster-Draper et al. [18] found that species with high wing loading and aspect ratio had higher activity in burned than untreated areas. Species with low wing loading and aspect ratio were not affected by prescribed fire in two studies in Florida and South Carolina [17, 21], but had lower activity in burned areas in a study in Ohio [20].

Although the frequency of large wildfires has increased [2], little is known about how these disturbances affect habitat selection by bats. Few studies have investigated the effects of wildfire on bats [22–26]. Buchalski et al. [24] found higher bat activity in burned versus unburned areas 1-year post fire, and Malison and Baxter [22] recorded greater bat activity in areas of high severity burn than in unburned areas. However, Jemison et al. [23] found significantly greater bat activity in unburned than burned areas. In areas that were burned, less severely burned areas had higher overall bat activity than high severity burned areas [23]. Law et al. [25] did not find a difference in bat activity between burned and unburned sites, and Steel et al. [26] found that most bat species in their study were positively affected by burn severity.

**Fig 1:**
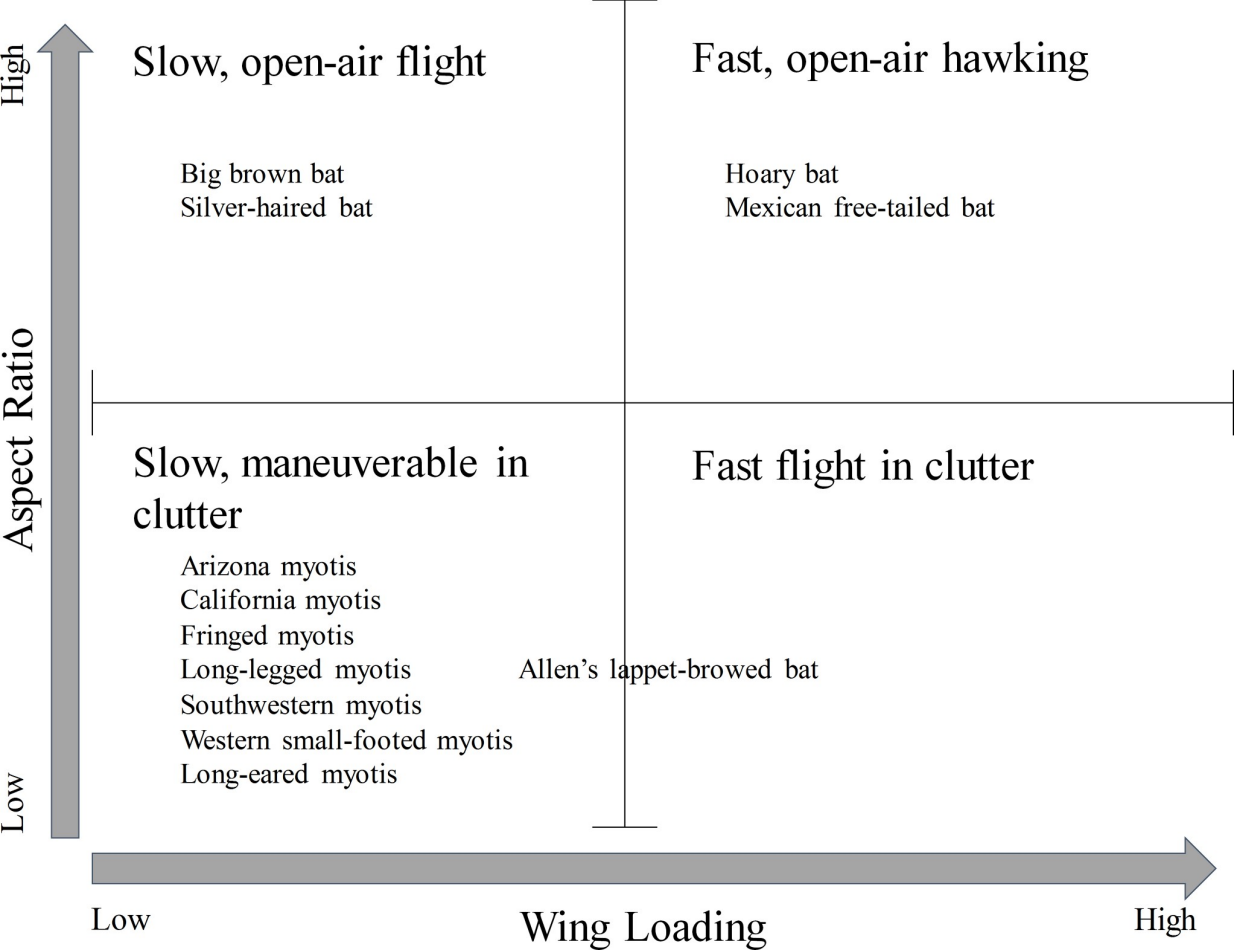
The relationship of wing loading to aspect ratio correlates to the predicted flight ecology of each species. The wing loading versus aspect ratio of potential bat species found in the Wallow fire perimeter, southeastern Arizona, USA. Figure adapted from Canals et al. [36].

Our objective was to assess how bats used the landscape at multiple spatial scales over varying fire severities 3 years post wildfire. Because water, landform type, elevation, vegetation type, and roads affect bat activity [27–31], we also assessed their influence on bat habitat use at multiple scales (fine [e.g., roosting] to coarse [e.g., foraging]). We chose to assess multiple scales to more accurately capture the spatial scales that are meaningful to the species of interest [32, 33]. This approach has been used for bats in other studies to more appropriately assess how the species uses its habitat (e.g., [34]). We predicted high activity of agile bats (e.g., low wing loading, low aspect ratio, high echolocation calls with a characteristic call frequency [F_c_] ≥33 kHz) in areas with no or low severity burn and high activity of fast-flying but less agile bats (high wing loading, high aspect ratio, and low echolocation calls with F_c_ <33 kHz) in areas of high severity burn (Fig 1) [35, 36]. We predicted high bat activity in areas with high water density, especially for agile, high frequency bats because these species generally will not fly as far in a night as less agile, low frequency bats (e.g., [37, 38], so they will stay closer to water. We also predicted that fast flying bats would respond at coarser landscape scales than agile bats that were better adapted to habitat use at fine, local scales since they will generally travel farther across the landscape in a night compared to more agile bats (Fig 1) (e.g., [37, 38]).

## Materials and methods

### Study area

**Fig 2:**
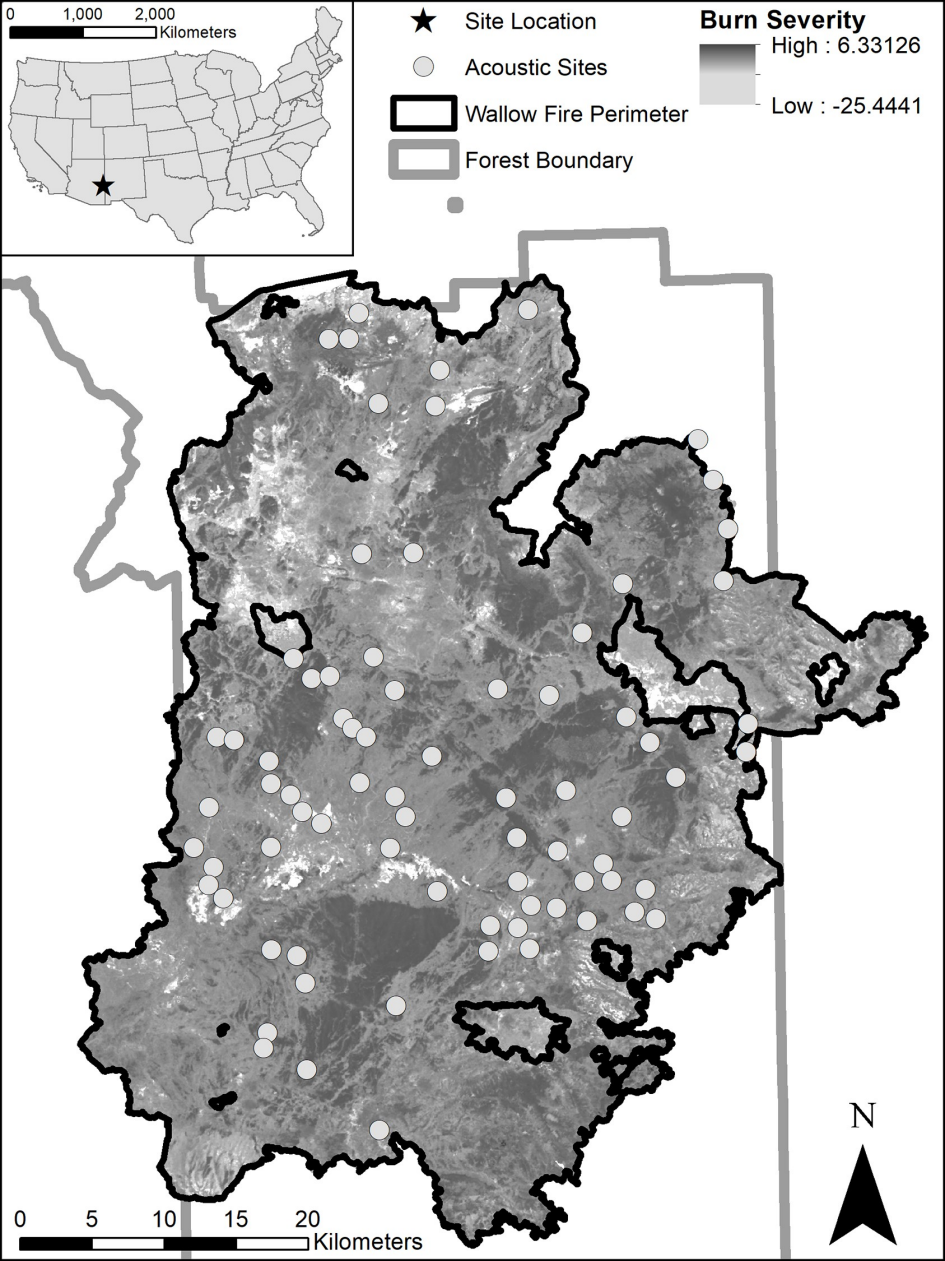
Study area showing burn severity and the boundary of the Wallow Fire in relation to the Apache-Sitgreaves National Forests and location in the United States. White dots represent the locations for placement of bat acoustic detectors in summer 2014. Burn severity of the Wallow Fire was determined using Composite Burn Index (CBI) values [42] which was determined using a relative differenced Normalized Burn Ratio (rdNBR) image from the Monitoring Trends in Burn Severity project [43].

We conducted our study from 11 June to 7 August 2014 within the perimeter of the Wallow Fire (Fig 2; NAD83, 12S 643903E, 3719106N) on the Apache-Sitgreaves National Forests in southeastern Arizona, USA. The Wallow Fire burned 217,721 ha in 2011 [39]. We surveyed bats in the ponderosa pine vegetation type [40], at sites that ranged in elevation from 2350 to 2690 m. Daily temperature and precipitation averaged 17.0 ± 0.3° C and 3.5 ± 1.1 mm, respectively, during the study period in the town of Alpine, 12 km from the center of the study area [41]. During the peak in precipitation in late summer (monsoon season; Jul–Aug), daily precipitation averaged 5.6 ± 2.1 mm in contrast to the pre-monsoon season in early summer (May–Jun) when it averaged 1.6 ± 0.8 mm.

### Acoustic recording

We placed SM3BAT acoustic detectors (Wildlife Acoustics, Maynard, Massachusetts, USA) at 4 points in each of 21 blocks (n = 84 points; Fig 2). Each block averaged ~5 km^2^ and consisted of one point in each of four burn severity classes, which we determined using the Rapid Assessment of Vegetation Condition after Wildfire (RAVG) layer [44]. This layer used immediate basal area (BA; the sum of the cross-section area of trees at breast height [45]) loss to determine burn severity [44]. Our four burn severity classes included 0–25%, 26–50%, 51–75%, and 76–100% BA loss within a 500 m radius from the acoustic detector point. We used ArcGIS 10.2.2 (ESRI, Redlands, CA, USA) to select points with the following criteria: ≥1 km from points in other blocks, ≥1 km from water sources (to avoid bias from bats drinking or foraging over water), ≥1 km from cliffs and buildings (to avoid bias from bats roosting in these structures), ≥50 m from roads to reduce risk of equipment loss or damage, and >50% of the area within a 500 m radius around each point was in one of the four burn severity classes.

We randomized blocks so that each point was surveyed twice for three consecutive nights each, once pre-monsoon (11 June to 11 July) and once during the monsoon season (12 July to 7 August). We used omnidirectional SM3-U1 microphones for each detector, which we connected to the SM3BAT detector with a 3 m cable and placed at the top of a 2.5 m pole. We tested each microphone to make sure that the sensitivity was similar across each detector unit. The microphone protected with a soft windscreen and was connected to the pole at a 45-degree angle so that water would drop off instead of pool in the microphone. We used four D-cell batteries to power each of the detectors. We deployed each detector in the center of a forest clearing ~30 m in diameter to avoid interference during acoustic recording from vegetative clutter. We recorded location, distance to forest edge (m), temperature (°C), relative humidity (%), and precipitation during deployment. We programmed detectors to record bat calls from sunset to sunrise, and during each 3-day period we concurrently surveyed two randomly-selected blocks.

Each detector recorded 5-sec .wav files when bats were detected with the microphone. We classified each 5-sec file with ≥2 pulses as a call sequence. We identified each echolocation call sequence using SonoBat 3.2.1 (SonoBat™, Arcata, CA, USA). In the SonoBat Batch Classify tool, we used a decision threshold of 0.95, acceptable call quality of 0.80, acceptable quality to tally passes of 0.20, and maximum number of calls to consider per file of 8. Since characteristic call frequencies overlapped for some species (Table 1), making differentiation unreliable at species level, we placed calls into five groups using SonoBat classification: low frequency (F_c_ <33 kHz), high frequency (F_c_ ≥33 kHz), *Myotis*, fringed myotis (*Myotis thysanodes*), and Mexican free-tailed bat (*Tadarida brasiliensis*) plus hoary bat (*Lasiurus cinereus*) calls (Table 1). Many bat acoustic studies use high and low frequency groups (e.g., [46, 47]), or put calls from the genus Myotis into one or two groups [e.g., [48, 49]) due to the difficulty in species discrimination. We felt confident in reliably separating fringed myotis from other *Myotis* species because of its low characteristic frequency (F_c_ <33 kHz) and short, broadband call compared to other species (e.g., [24]). We felt confident in separating Mexican free-tailed bat and hoary bat from other low frequency species because of their lower characteristic frequencies and call shape, but we were not confident in separating those two species. However, both species are open-air flyers with high wing loading and aspect ratio (Table 1). We averaged calls per hour by group for each point for each 3-day period. The average calls per hour for each of the groups were our response variables.

**Table 1:** Arizona bat species, call characteristics, and morphology. Scientific name, common name, range in characteristic call frequency (F_c_), call category (LF = low frequency, Tb/Lc = Mexican free-tailed bat [*Tadarida brasiliensis*]/hoary bat [*Lasiurus cinereus*], HF = high frequency, M = Myotis, Myth = fringed myotis [*Myotis thysanodes*]), aspect ratio, wing loading, and our prediction of the response to increasing burn severity (Prediction; + = positive response,— = negative response) for bat species occurring in ponderosa pine forests on the Apache-Sitgreaves National Forests, Arizona, USA.

Scientific name	Common name	F_c_ range (kHz)^a^	Call category	Aspect ratio	Wing loading	Prediction
*Myotis californicus*	California myotis	46–52	HF, M	low	low	-
*Myotis ciliolabrum*	western small-footed myotis	41–45	HF, M	low	low	-
*Myotis volans*	long-legged myotis	40–44	HF, M	low	low	-
*Myotis occultus*	Arizona myotis	40–43	HF, M	low	low	-
*Myotis auriculus*	southwestern myotis	33–45	HF, M	low	low	-
*Myotis evotis*	long-eared myotis	33–37	HF, M	low	low	-
*Eptesicus fuscus*	big brown bat	27–31	LF	high	low	+
*Idionycteris phyllotis*	Allen’s lappet-browed bat	25–29^b^	LF	low	intermediate	+
*Lasionycteris noctivagans*	silver-haired bat	25–28	LF	high	low	+
*Tadarida brasiliensis*	Mexican free-tailed bat	23–28	LF, Tb/Lc	high	high	+
*Myotis thysanodes*	fringed myotis	21–28	LF, M, Myth	low	low	-
*Lasiurus cinereus*	hoary bat	19–24	LF, Tb/Lc	high	high	+

^a^ J.M. Szewczak, Humboldt State University Bat Lab (personal communication).

^b^ [50]

### Landscape variables

We used a moving window analysis in ArcGIS 10.2.2 (ESRI, Redlands, CA, USA) to determine values for burn severity, elevation, range in elevation, land cover, landform type, stream density, lake density, and road density around each point at 7 scales (90, 180, 360, 720, 1440, 2880, and 5760 m radius; Table 2). We selected these scales because they represented local (e.g., 90 to 720 m) to landscape (≥1440 m) habitat use by bats [e.g., 34, 51]. We obtained Composite Burn Index (CBI) values [42] post-fire to determine burn severity using a relative differenced Normalized Burn Ratio (rdNBR) image of the Wallow Fire area from the Monitoring Trends in Burn Severity project [MTBS; 43]. This provided a continuous value for burn severity. We used the National Land Cover Database [52] to determine land cover. We reclassified land cover into 2 categories: evergreen forest and grassland/shrub/scrub where evergreen forest was used as the reference category. We combined grassland, shrub, and scrub land covers because they were all open land cover types and, individually, they did not make up a large portion of the landscape. We used a landform data layer that consisted of 15 landform classes to determine the landform type [53]. We reclassified landform types into 2 categories: upper slopes/ridge and lower slopes/valley where upper slopes/ridge was used as the reference category. We calculated lake, stream, and road densities using data from the Apache-Sitgreaves National Forests [54]. All continuous values were standardized to z-scores before analysis, and we tested variables for collinearity in program R with Spearman’s rho statistic.

**Table 2:** Variables used in analysis of bat acoustic data. Landscape variables used in the analysis. Variable, type (CO = continuous or CA = categorical), range or percent of area covered, and a description of each variable are provided.

Variable	Type^a^	Range or % of Area	Description
Burn severity	CO	-3.77 to 3.00	An RdNBR image that measures change relative to pre-fire vegetation. Uses Composite Burn Index (CBI) values where <0 means no change and >2.25 means high severity [42].
Elevation Range	CO	1.43 m to 1017.73 m	Used a digital elevation model (DEM) to calculate the difference between highest and lowest point at 90, 180, 360, 720, 1440, 2880, 5760 m.
Stream Density	CO	0.00 m/m^2^ to 7.29 m/m^2^	Stream data obtained from United States Forest Service (USFS) Apache-Sitgreaves National Forests.
Lake Density	CO	0.00 m/m^2^ to 0.06 m/m^2^	Lake data obtained from US Department of Interior.
Road Density	CO	0.00 m/m^2^ to 9.81 m/m^2^	Obtained combining road data from USFS Apache-Sitgreaves National Forests and TIGER/Line from the US Census Bureau.
Landcover	CA		NLCD 2011 from [52]
Evergreen Forest		73.4	
Grassland/Shrub/Scrub		25.6	
Landform	CA		From [53]
Upper slopes/ridge		49.9	
Lower slopes/valley		45.2	

^a^CO = continuous variable, CA = categorical variable

### Statistical analyses

We used a 2-step process to predict response of each bat group to covariates at the most appropriate scale. We first performed a univariate analysis to determine the best fitting scale for each landscape variable. We used linear mixed model regression with season (i.e., pre-monsoon or post-monsoon) as a random variable in program R using package lme4 (R statistical program v. 3.3.3). We ran 7 linear mixed models for each landscape variable in combination with each species group to get the most appropriate scale for each landscape variable and species group [e.g., 34]. The scale with the lowest *P* value for each variable was used in additional modeling; variables that did not have a scale with a *P* value <0.20 were dropped from the analysis [e.g., 34]. In the second step, we performed multivariate analysis using the variables at their most appropriate scales for each species group to create a list of candidate models (S1 Appendix). We used the package lme4 in program R (R statistical program v. 3.3.3) to run general linear mixed model regressions with season as a random variable for the multivariate analysis. All models with ΔAICc ≤4 were averaged in R using package MuMIn (R statistical program v. 3.3.3) to obtain the coefficients, standard errors, and relative variable importance for each covariate [55, 56]. Covariates with a relative variable importance value ≥0.50 had a strong species response, <0.50 and ≥0.30 had a moderate species response, and <0.30 had a weak species response [e.g., 7, 57].

We created maps estimating the average number of calls per night for each species group in the study area using the Raster Calculator tool in ArcGIS 10.2.2 (ESRI, Redlands, CA, USA). For each species group, we created the map using the averaged estimates of variables that had an importance value of ≥0.30.

## Results

**Fig 3:**
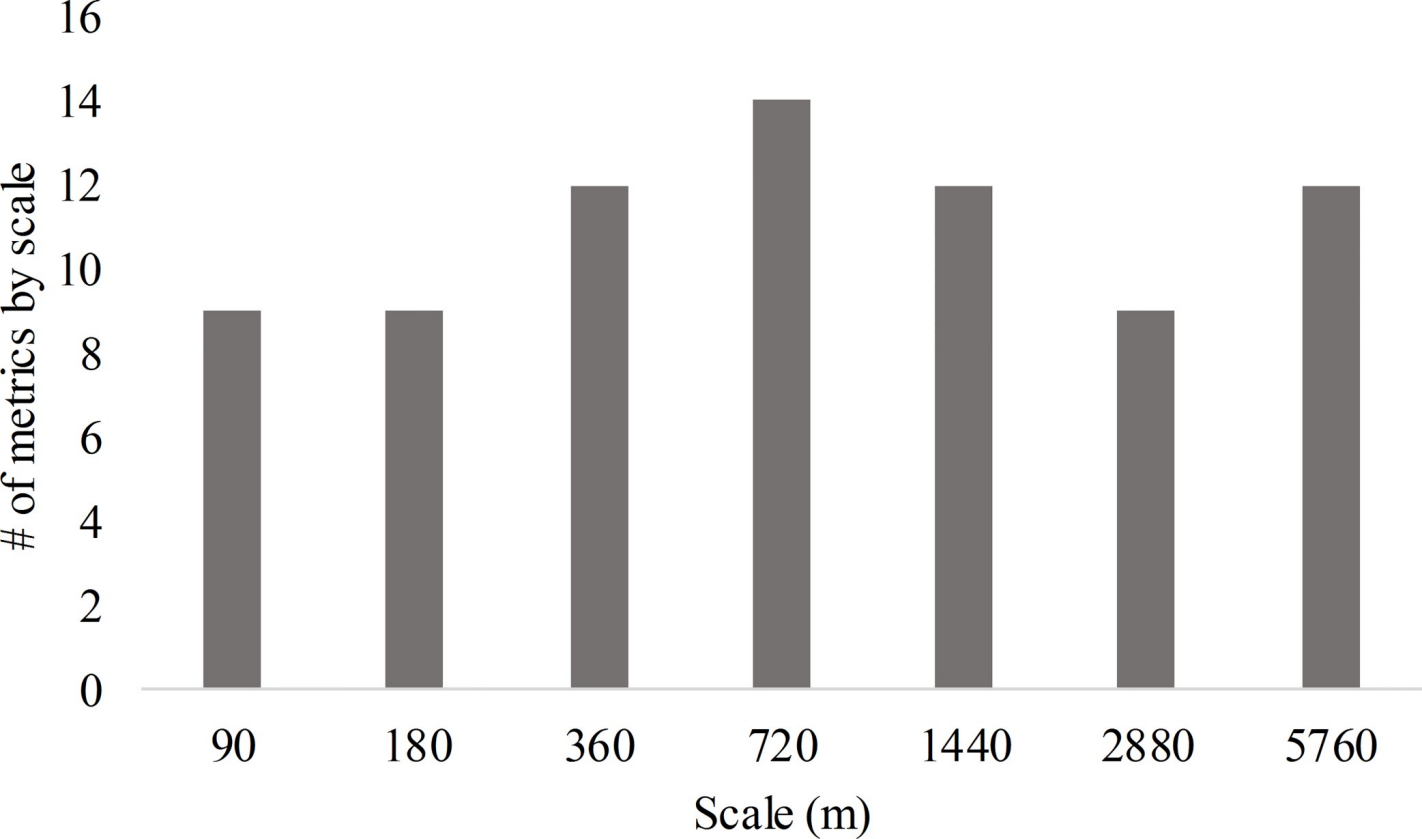
Selection of spatial scales in models. The number of times that each spatial scale was selected as the most appropriate scale in the univariate linear mixed model analysis.

We collected and classified 63,990 files as bat calls. Of these, 69, 31, 7, 1, and 14%, respectively, were classified as low frequency, high frequency, *Myotis*, fringed myotis, and Mexican free-tailed bat/hoary bat. In the univariate analysis, we found no scale was selected preferentially, therefore scales varied in the multivariate models (Fig 3).

Competitive models consisted of covariates ranging from fine to coarse scales. Scales selected for the low frequency and high frequency groups ranged from 360 m to 5760 m and 90 to 5760 m, respectively (Table 3, Fig 3). Scales selected for *Myotis*, fringed myotis, and Mexican free-tailed bat/hoary bat ranged from 180 m to 5760 m, 90 m to 2880 m, and 90 m to 5760 m, respectively (Table 3, Fig 3).

**Table 3:** Variables included in models for each species group. Variables included in the models for each bat species group (low frequency, high frequency, *Myotis*, fringed myotis [*Myotis thysanodes*], and Mexican free-tailed bat [*Tadarida brasiliensis*]/hoary bat [*Lasiurus cinereus*]), the selected scale for each variable, estimate, unconditional standard error (SE), and variable importance. Evergreen forest was used as the reference category for Landcover, and Upper slopes/ridges was used as the reference category for Landform.

Species Group	Variable	Scale (m)	Estimate	SE	Importance
Low frequency	Stream density	5760	6.042	3.847	0.86
	Elevation range	720	2.009	2.083	0.64
	Burn severity	5760	0.737	1.741	0.34
	Intercept		4.876	5.601	
High frequency	Elevation range	2880	1.549	1.433	0.69
	Landcover				
	Shrub/scrub/grassland	90	-2.031	2.186	0.61
	Road density	5760	-1.244	1.558	0.56
	Landform				
	Lower slopes/valleys	360	1.026	1.321	0.53
	Burn severity	90	-0.320	0.673	0.36
	Intercept		6.893	2.134	
*Myotis*	Landform				
	Lower slopes/valleys	360	0.845	0.296	1.00
	Stream density	180	-0.167	0.150	0.69
	Road density	5760	-0.443	0.431	0.66
	Landcover				
	Shrub/scrub/grassland	180	-0.484	0.515	0.62
	Elevation range	5760	0.379	0.409	0.61
	Intercept		1.594	0.623	
Fringed myotis	Burn severity	2880	-0.189	0.102	0.90
	Lake density	2880	0.108	0.067	0.86
	Stream density	720	0.093	0.071	0.77
	Elevation range	90	-0.105	0.085	0.75
	Intercept		0.174	0.103	
Mexican free-tailed bat/hoary bat	Stream density	5760	1.339	1.087	0.76
	Elevation range	360	0.708	0.595	0.74
	Burn severity	90	0.476	0.521	0.61
	Landcover				
	Shrub/scrub/grassland	90	0.775	1.130	0.47
	Road density	180	0.115	0.191	0.41
	Landform				
	Lower slopes/valleys	720	0.120	0.403	0.26
	Intercept		0.609	1.685	

**Fig 4:**
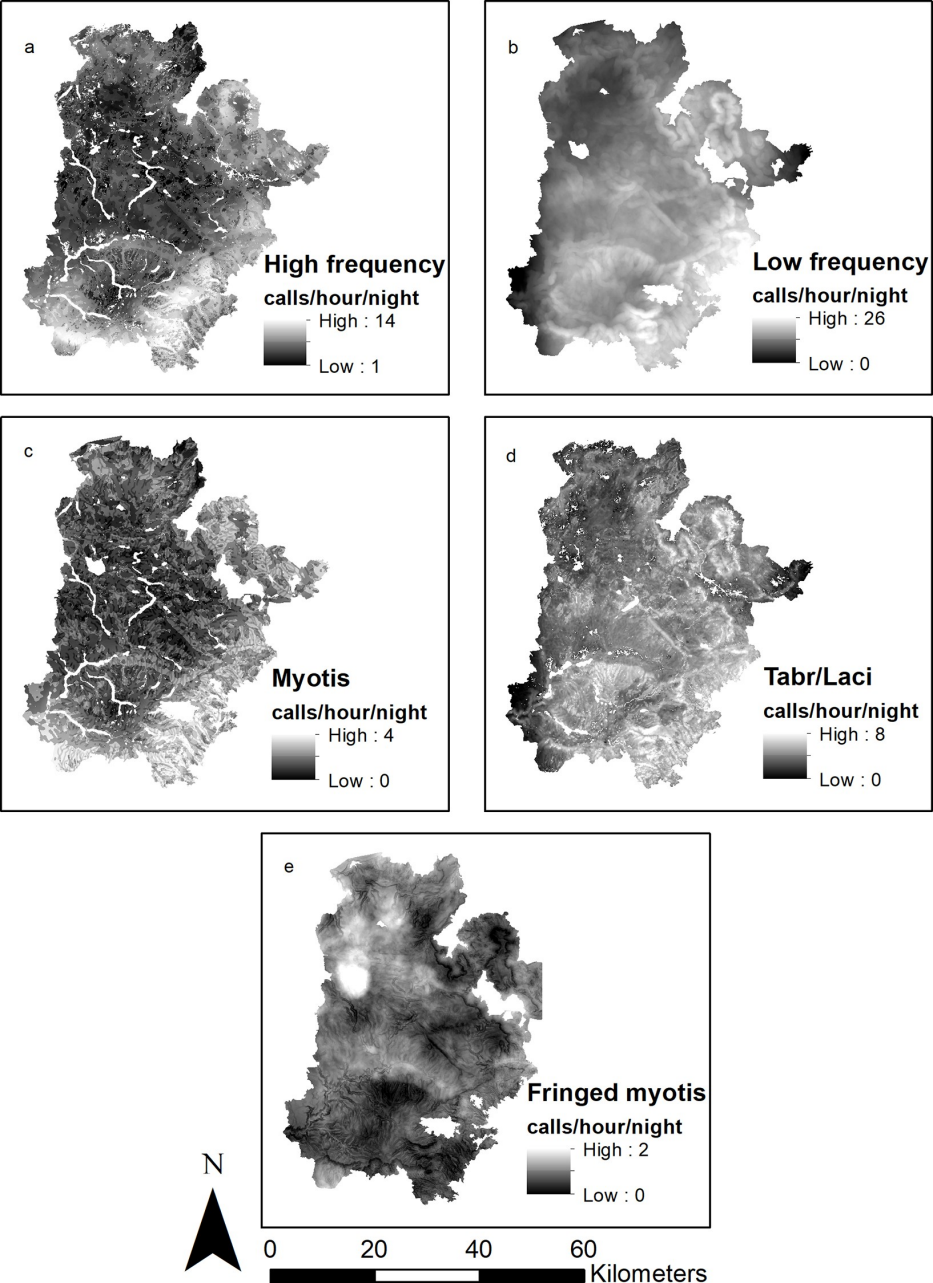
Estimated activity of bats in the Wallow Fire burn area three years post-fire. Estimated bat activity (number of echolocation calls/hour/night) of: a. low frequency (characteristic frequency [F_c_] <33 kHz) bats, b. high frequency (characteristic frequency [Fc] >33 kHz) bats, c. *Myotis*, d. Mexican free-tailed (*Tadarida brasiliensis*) and hoary bats (*Lasiurus cinereus*; [Tabr/Laci]), and e. fringed myotis (*Myotis thysanodes*).

The group consisting of low frequency calls responded positively and strongly to stream density and elevation range, and positively and moderately to burn severity (Table 3). Bats in this group selected areas of high burn severity with greater range in elevation and higher stream density (Fig 4a).

Bats with high frequency calls had a strong response to elevation range, landcover, road density, and landform, and responded moderately to burn severity (Table 3). This group responded positively to range in elevation but negatively to burn severity and road density. Bats with high frequency calls selected evergreen forest over shrub/scrub/grassland landcover and lower slopes/valleys over upper slopes/ridges for landform (Fig 4b).

Calls classified as *Myotis* had a strong response to landform, stream density, road density, landcover, and elevation range (Table 3). *Myotis* responded positively to elevation range and negatively to stream density and road density. This group selected lower slopes/valleys over upper slopes/ridges for landform and selected evergreen forest over shrub/scrub/grassland landcover (Fig 4c).

Mexican free-tailed and hoary bats responded strongly to stream density, elevation range, and burn severity, moderately to landcover and road density, and weakly to landform (Table 3). These species responded positively to stream density, elevation range, burn severity, and road density. This group selected shrub/scrub/grassland landcover over evergreen forest and lower slopes/valleys over upper slopes/ridges (Fig 4d).

Fringed myotis had a strong response to burn severity, lake density, stream density, and elevation range (Table 3). Fringed myotis responded positively to lake density and stream density but negatively to burn severity and elevation range (Fig 4e).

## Discussion

Burn severity was important in predicting bat activity in a post wildfire landscape for one species, but other landscape variables were more important for predicting activity of bat groups that included ≥2 species. Fringed myotis is a small-bodied bat with low aspect ratio and low wing loading, and our results suggest that this species avoids large areas of high severity burn. Jemison et al. [23] also found that small-bodied bats with low aspect ratio and wing loading were negatively affected by wildfire compared to species with big bodies and long, narrow wings. Bats with lower wing loading and a lower aspect ratio are better suited to maneuvering around and gleaning prey from objects than species with high wing loading and aspect ratio [35, 58, 59]. However, Buchalski et al. [24] found the opposite with *Myotis* activity higher in areas of high burn severity. Buchalski et al. [24] conducted their study in areas with only moderate- to high-severity burn one-year post fire in mixed conifer forest. Jemison et al. [23] conducted their study two-years post fire, and our study was three-years post fire. Some species of insects increase in abundance directly after a fire [60, 61] therefore increasing prey density in burned areas for bats the year immediately after the fire. Brigham et al. [62] found that small-bodied, maneuverable bat species would still use open habitat if that habitat was higher in prey density than more closed habitat. Morphology and call frequency can predict habitat selection, but bats use the landscape differently over time depending on food availability.

Burn severity on the landscape helped to predict activity for one bat species, but other landscape variables were key for other bat species groups. Water density was more important than burn severity for most bats in our study. Riparian ecosystems are uncommon in the western United States (e.g., only 0.4% of the land base in Arizona; [63]). Reproductive females especially need water and had higher capture rates at water sources than males or non-reproductive females [27, 28, 64]. Generally, higher stream density on the landscape benefited bats in our study area, through either increased prey density or availability of drinking water. However, *Myotis* group selected against increasing stream density at a local scale. At more local spatial scales, stream density may be less important because many streams in this area are intermittent, often fast moving, or covered with vegetation. Bats fly over still water to drink during flight and avoid running water, presumably because of the difficulty and risk of obtaining water from streams or rivers while drinking on the wing [65–67]. Human-modified water sources, such as livestock ponds, are common in the southwest and might be more important than natural streams in this area because they are more reliable and non-flowing [68, 69]. *Myotis* are small and have a higher surface area to weight ratio than other bat species in our study. These small bats need reliable water sources to remain hydrated in this arid environment.

Selection for lower slopes and valleys could be associated with elevation range and temperature regulation for reproductive females [70, 71]. There was high activity of agile flyers when there was a greater range in elevation over a coarse landscape scale. When females care for young during the summer, they need to roost at lower, warmer elevations, but may choose to drink and forage at higher elevations. In the Black Hills and in parts of the eastern United States, reproductive females were captured less often at high elevations than low elevations compared to males and non-reproductive females [27, 70, 71]. There were also fewer reproductive females captured at high elevations than low elevations compared to males and non-reproductive females in our study area [64]. A range in elevation on the landscape can support reproductive females as well as non-reproductive females and males. However, we found that one species, the fringed myotis, selected against elevation range at a fine scale. Although *Myotis* move between roosts frequently [72], fringed myotis do not move far when changing roosts, showing fidelity to a roost area and not to a particular roost tree [73–75]. Fringed myotis may be searching for roosting opportunities in close proximity to other roosts, which could facilitate the movement of young to new roosts [76]. Fringed myotis was our only single-species group, and the diverse *Myotis* group could have selected a wide range in elevation because it consists of more than one species where each species uses a different range in elevation. Because we grouped all *Myotis* together, the overall range in elevation becomes important.

Selection of a range in elevation may also be related to food availability. McCoy [77] found higher species richness of insects at mid elevations in the United States, suggesting that a greater range in elevation provided more availability for foraging for insectivorous bats. Insects may also be more abundant close to water sources [e.g., 62, 27], meaning more prey for bats in riparian areas. Insects were influenced by cattle grazing in southeastern Arizona and were less abundant in grazed grasslands [78]. This may be similar for our study area and grazed grasslands/shrub/scrub provided less prey for bats than evergreen forest.

Roads provided some bat species with travel and foraging areas but other species avoided roads. Forest roads provide open habitat for low frequency bats to use as flight corridors or as open areas around a roost in an otherwise forested area [e.g., 31], and in our study low frequency bats had high activity in areas of high road density at a fine scale. Smaller species that echolocate at higher frequencies, like *Myotis*, responded negatively to road density, which suggests that these species avoid open spaces such as roads. *Myotis* and other clutter-adapted species fly closer to the ground over roads than high, open-air flying species and are more likely to be killed at roads [79, 80]; roads cause barriers to some bats because of habitat fragmentation [81, 82].

Important relationships in how a species selects its habitat will be missed if only one spatial scale is considered [83–85]. For bat groups in our study, no scale proved best; the habitat element of interest affected scale importance. This was also true for the single species group in our study, fringed myotis. This indicates that other bat species could also respond to habitat elements across a range of scales. This variability in scale may be attributed to the mobility of bats; they use habitat elements at scales ranging from roost to foraging area. A heterogeneous landscape increases roosting opportunities and variability in foraging opportunities [86].

Studying bats with acoustic devices is less labor intensive than capturing animals, can effectively sample bats that fly above the height of most nets (>7.8 m; [87, 88]), detect individuals that learn to avoid nets [89–91], and sample more efficiently (e.g., more locations can be sampled than with capture techniques). However, habitat structure [92], temperature, and humidity [93] can affect how sound moves in the environment, which influences detectability of the bat call [88], and acoustics cannot determine individual information (i.e., sex, age group, reproductive condition, species for locations like the southwest United States) or abundance. Despite these limitations, our study is one of few that identified responses of bats to wildfire.

## Conclusions

We found that multiple spatial scales were important in determining habitat use. Managing forests for heterogeneity benefits a diversity of bat species. Although burn severity did negatively affect one bat species, water and elevation range were most important, at least in the immediate post-fire years. We might expect the importance of habitat elements such as water (e.g., water use) to differ in other ecosystem types since our study took place in an arid environment. In areas where water is more abundant, burn severity might have a greater effect. Burn severity could also be more important for individual species, and grouping species together in analyses reduces the importance of burn severity. However, maintaining and protecting slow-moving water, like livestock ponds, is important for bats, especially in the arid western United States. Some species groups opportunistically used forest roads, so although some bats responded negatively to roads, roads were not always negative for other bats. Landscape scales of ≥5760 m should especially be considered when managing habitat for bats that can fly long distances nightly from roosts to foraging areas. The largest spatial scale should be the home range or maximum nightly flight distance for the bat species of interest.

## Supporting information

S1 Appendix Candidate model list. The log likelihood (LogLike), AICc, ΔAICc, and weight (*w*_*i*_) for each of the candidate models with a ΔAICc ≤4 and the null model for each species group.
(DOCX)
