# Supplementary material for: Water and elevation are more important than burn severity in predicting bat activity at multiple scales in a post-wildfire landscape
Source: PLoS One. 2020 Apr 8;15(4):e0231170. doi: 10.1371/journal.pone.0231170 (PMC7141652; doi:10.1371/journal.pone.0231170)
Supplement: S1 Appendix — The log likelihood (LogLike), AICc, ΔAICc, and weight (wi) for each of the candidate models with a ΔAICc ≤4 and the null model for each species group. (DOCX) [file pone.0231170.s001.docx]

S1 Appendix. Candidate model list. The log likelihood (LogLike), AICc, ∆AICc, and weight (*w_i_*) for each of the candidate models with a ∆AICc ≤4 and the null model for each species group.

| Models^a^ | | LogLike | AICc | *∆*AICc | *w_i_* |
| --- | --- | --- | --- | --- | --- |
| Low Frequency | |  |  |  |  |
|  | elev720, stream5760 | -470.25 | 951.00 | 0.00 | 0.33 |
|  | stream5760 | -471.80 | 952.00 | 0.93 | 0.20 |
|  | burn5760, elev720, stream5760 | -470.01 | 952.80 | 1.74 | 0.14 |
|  | burn5760, stream5760 | -471.21 | 953.00 | 1.93 | 0.12 |
|  | elev720 | -472.73 | 953.80 | 2.78 | 0.08 |
|  | burn5760, elev720 | -472.09 | 954.70 | 3.68 | 0.05 |
|  | NULL | -474.61 | 955.40 | 4.40 | 0.04 |
| High Frequency | |  |  |  |  |
|  | elev2880,landform360,veg90 | -391.82 | 796.40 | 0.00 | 0.09 |
|  | elev2880,veg90 | -393.10 | 796.70 | 0.33 | 0.07 |
|  | landform360,road5760,veg90 | -392.06 | 796.90 | 0.48 | 0.07 |
|  | elev2880,landform360,road5760,veg90 | -391.13 | 797.30 | 0.87 | 0.06 |
|  | road5760,veg90 | -393.37 | 797.30 | 0.88 | 0.06 |
|  | elev2880,road5760,veg90 | -392.33 | 797.40 | 1.02 | 0.05 |
|  | burn90,elev2880 | -393.60 | 797.70 | 1.33 | 0.05 |
|  | elev2880,landform360 | -393.65 | 797.80 | 1.43 | 0.04 |
|  | burn90,elev2880,landform360 | -392.54 | 797.80 | 1.44 | 0.04 |
|  | burn90,elev2880,landform360,veg90 | -391.51 | 798.00 | 1.63 | 0.04 |
|  | burn90,elev2880,veg90 | -392.66 | 798.10 | 1.68 | 0.04 |
|  | elev2880 | -394.89 | 798.10 | 1.73 | 0.04 |
|  | landform360,road5760 | -393.90 | 798.30 | 1.93 | 0.03 |
|  | road5760 | -395.17 | 798.70 | 2.30 | 0.03 |
|  | elev2880,landform360,road5760 | -393.05 | 798.80 | 2.45 | 0.03 |
|  | burn90,landform360,road5760,veg90 | -391.95 | 798.90 | 2.51 | 0.03 |
|  | elev2880,road5760 | -394.22 | 799.00 | 2.58 | 0.02 |
|  | burn90,landform360,road5760 | -393.15 | 799.00 | 2.65 | 0.02 |
|  | burn90,elev2880,road5760 | -393.18 | 799.10 | 2.71 | 0.02 |
|  | burn90,road5760 | -394.29 | 799.10 | 2.71 | 0.02 |
|  | burn90,road5760,veg90 | -393.19 | 799.10 | 2.73 | 0.02 |
|  | burn90,elev2880,road5760,veg90 | -392.08 | 799.20 | 2.77 | 0.02 |
|  | burn90,elev2880,landform360,road5760,veg90 | -390.96 | 799.20 | 2.83 | 0.02 |
|  | burn90,elev2880,landform360,road5760 | -392.15 | 799.30 | 2.92 | 0.02 |
|  | landform360,veg90 | -394.72 | 800.00 | 3.57 | 0.02 |
|  | NULL | -397.83 | 801.90 | 5.48 | 0.01 |
| Myotis thysanodes | |  |  |  |  |
|  | burn2880,elev90,landform2880,stream720 | -76.36 | 167.70 | 0.00 | 0.35 |
|  | burn2880,landform2880,stream720 | -78.18 | 169.10 | 1.39 | 0.18 |
|  | burn2880,elev90,landform2880 | -78.63 | 170.00 | 2.29 | 0.11 |
|  | elev90,landform2880,stream720 | -78.88 | 170.50 | 2.78 | 0.09 |
|  | burn2880,elev90,stream720 | -78.97 | 170.70 | 2.96 | 0.08 |
|  | burn2880,elev90 | -80.58 | 171.70 | 3.97 | 0.05 |
|  | burn2880,landform2880 | -80.59 | 171.70 | 3.98 | 0.05 |
|  | NULL | -88.10 | 182.40 | 14.68 | 0.00 |
| Mexican free-tailed bat and hoary bat | |  |  |  |  |
|  | burn90,elev360,stream5760 | -312.53 | 637.80 | 0.00 | 0.07 |
|  | elev360,road180,stream5760,veg90 | -311.74 | 638.50 | 0.68 | 0.05 |
|  | elev360,stream5760,veg90 | -312.87 | 638.50 | 0.69 | 0.05 |
|  | burn90,elev360 | -314.13 | 638.80 | 0.99 | 0.04 |
|  | burn90,elev360,road180,stream5760 | -311.90 | 638.80 | 1.01 | 0.04 |
|  | burn90,elev360,stream5760,veg90 | -311.97 | 638.90 | 1.14 | 0.04 |
|  | burn90,elev360,road180 | -313.22 | 639.20 | 1.38 | 0.04 |
|  | elev360,stream5760 | -314.33 | 639.20 | 1.39 | 0.03 |
|  | stream5760,veg90 | -314.35 | 639.20 | 1.44 | 0.03 |
|  | burn90,stream5760,veg90 | -313.29 | 639.30 | 1.53 | 0.03 |
|  | elev360,road180,stream5760 | -313.33 | 639.40 | 1.62 | 0.03 |
|  | burn90,elev360,road180,stream5760,veg90 | -311.17 | 639.60 | 1.84 | 0.03 |
|  | burn90,elev360,landform720,stream5760 | -312.36 | 639.70 | 1.92 | 0.03 |
|  | burn90,stream5760 | -314.61 | 639.70 | 1.94 | 0.03 |
|  | road180,stream5760,veg90 | -313.55 | 639.90 | 2.05 | 0.03 |
|  | burn90,elev360,landform720 | -313.79 | 640.30 | 2.53 | 0.02 |
|  | burn90,road180,stream5760,veg90 | -312.78 | 640.60 | 2.77 | 0.02 |
|  | burn90,landform720,stream5760 | -313.92 | 640.60 | 2.80 | 0.02 |
|  | burn90,elev360,landform720,road180 | -312.81 | 640.60 | 2.82 | 0.02 |
|  | elev360,landform720,road180,stream5760,veg90 | -311.66 | 640.60 | 2.83 | 0.02 |
|  | burn90,elev360,landform720,road180,stream5760 | -311.67 | 640.70 | 2.86 | 0.02 |
|  | elev360,landform720,stream5760,veg90 | -312.83 | 640.70 | 2.88 | 0.02 |
|  | elev360,road180 | -315.10 | 640.70 | 2.94 | 0.02 |
|  | burn90,elev360,veg90 | -314.00 | 640.80 | 2.96 | 0.02 |
|  | burn90 | -316.27 | 640.90 | 3.08 | 0.02 |
|  | burn90,elev360,road180,veg90 | -312.95 | 640.90 | 3.11 | 0.02 |
|  | burn90,landform720,stream5760,veg90 | -312.97 | 641.00 | 3.16 | 0.01 |
|  | landform720,stream5760,veg90 | -314.13 | 641.00 | 3.22 | 0.01 |
|  | elev360,road180,veg90 | -314.15 | 641.10 | 3.25 | 0.01 |
|  | burn90,elev360,landform720,stream5760,veg90 | -311.88 | 641.10 | 3.27 | 0.01 |
|  | burn90,landform720 | -315.28 | 641.10 | 3.29 | 0.01 |
|  | elev360,landform720,stream5760 | -314.21 | 641.20 | 3.36 | 0.01 |
|  | elev360,landform720,road180,stream5760 | -313.14 | 641.30 | 3.49 | 0.01 |
|  | burn90,road180,stream5760 | -314.36 | 641.50 | 3.66 | 0.01 |
|  | landform720,road180,stream5760,veg90 | -313.23 | 641.50 | 3.66 | 0.01 |
|  | elev360 | -316.63 | 641.60 | 3.80 | 0.01 |
|  | burn90,elev360,landform720,road180,stream5760,veg90 | -311.05 | 641.80 | 3.95 | 0.01 |
|  | NULL | -320.03 | 646.30 | 8.46 | 0.00 |
| Myotis | |  |  |  |  |
|  | landform360,road5760,stream180,veg180 | -218.41 | 451.80 | 0.00 | 0.17 |
|  | elev5760,landform360,stream180,veg180 | -218.79 | 452.60 | 0.75 | 0.12 |
|  | elev5760,landform360,road5760,stream180,veg180 | -217.76 | 452.80 | 0.99 | 0.10 |
|  | landform360,road5760,stream180 | -220.08 | 452.90 | 1.08 | 0.10 |
|  | elev5760,landform360,stream180 | -220.12 | 453.00 | 1.16 | 0.09 |
|  | elev5760,landform360,veg180 | -220.46 | 453.70 | 1.84 | 0.07 |
|  | elev5760,landform360,road5760,stream180 | -219.34 | 453.70 | 1.86 | 0.07 |
|  | landform360,road5760,veg180 | -220.47 | 453.70 | 1.86 | 0.07 |
|  | elev5760,landform360,road5760,veg180 | -219.59 | 454.20 | 2.35 | 0.05 |
|  | elev5760,landform360 | -222.01 | 454.60 | 2.72 | 0.04 |
|  | landform360,road5760 | -222.40 | 455.30 | 3.49 | 0.03 |
|  | elev5760,landform360,road5760 | -221.38 | 455.50 | 3.69 | 0.03 |
|  | NULL | -229.95 | 466.10 | 14.28 | 0.00 |

^a^ Variables for the models included: burn = burn severity, elev = elevation range, landform = landform type, road = road density, stream = stream density, veg = landcover type. The number following the variable name is the scale (in meters) that was selected as the most appropriate scale for that variable for that species group in the univariate linear mixed modeling.
